# A Novel Approach of Identifying Immunodominant Self and Viral Antigen Cross-Reactive T Cells and Defining the Epitopes They Recognize

**DOI:** 10.3389/fimmu.2018.02811

**Published:** 2018-12-03

**Authors:** Junbao Yang, Lichen Jing, Eddie A. James, John A. Gebe, David M. Koelle, William W. Kwok

**Affiliations:** ^1^Benaroya Research Institute at Virginia Mason, Seattle, WA, United States; ^2^Department of Medicine, University of Washington, Seattle, WA, United States; ^3^Vaccine and Infectious Diseases Division, Fred Hutchinson Cancer Research Center, Seattle, WA, United States

**Keywords:** molecular mimicry, T cell cross-reactivity, influenza, glutamate decarboxylase 65, autoreactive T cells

## Abstract

Infection and vaccination can lead to activation of autoreactive T cells, including the activation of cross-reactive T cells. However, detecting these cross-reactive T cells and identifying the non-self and self-antigen epitopes is difficult. The current study demonstrates the utility of a novel approach that effectively accomplishes both. We utilized surface expression of CD38 on newly activated CD4 memory T cells as a strategy to identify type 1 diabetes associated autoreactive T cells activated by influenza vaccination in healthy subjects. We identified an influenza A matrix protein (MP) specific CD4+ T cell clone that cross-recognizes an immunodominant epitope from Glutamic Acid Decarboxylase 65 (GAD65) protein. The sequences of the MP and GAD65 peptides are rather distinct, with only 2 identical amino acids within the HLA-DR binding region. This result suggests that activation of autoreactive T cells by microbial infection under certain physiological conditions can occur amongst peptides with minimum amino acid sequence homology. This novel strategy also provides a new research pathway in which to examine activation of autoreactive CD4+ T cells after vaccination or natural infection.

## Introduction

Type 1 diabetes (T1D) is an autoimmune disease in which islet beta cells are selectively destroyed. Both genetic and environmental factors contribute to the onset of disease (1, 2). Genetic factors associated with disease susceptibility have been well studied; the HLA class II locus and other loci encoding immune regulatory molecules top the list of genetic contributors (3, 4). These genetic associations support a crucial role for CD4 T cells in initiating and maintaining beta cell autoimmunity. In contrast, environmental factors that contribute to the pathogenesis of T1D remain elusive. In recent decades, there has been a rapid rise in incidence of T1D worldwide (5), with nations reporting up to a 3.9% average annual increase for more than 15 years (6). This trend emphasizes a need to identify the environmental factors that influence T1D pathogenesis. Activation of self-antigen responsive (autoreactive) T cells by viral infection through epitope molecular mimicry is suggested to be a possible mechanism (7). However, many technical challenges, including difficulties in detecting autoreactive T cells, have hampered progress in this field. Recently, we applied a modified CD154 up-regulation assay (8–11) to detect rare auto-reactive T cells *ex vivo* and identify their epitope specificity. Using these approaches and applying what we already know about antigenic epitopes within influenza A and islet antigens, we have developed a novel strategy to identify not only the cross-reactive T cells but also the mimicking viral- and self-antigen epitopes.

This strategy takes advantage of the observation that CD38 is upregulated on memory CD4+ T cells following activation (12, 13). Specifically, resting memory influenza specific CD4+ T cells are CD38-, but become CD38 bright in the periphery starting 7–14 days after influenza vaccination or infection (14). Cell surface expression of CD38 in influenza specific cells remains upregulated for more than a month following vaccination but, declines to basal levels in about 2 months after antigen clearance (11, 14). This observation indicates that CD38 expression on memory CD4+ T cells is a marker of their recent activation *in vivo*.

We postulated that autoreactive islet antigen specific T cells that can cross react to influenza antigen will also up-regulate CD38 upon influenza vaccination, allowing a means of effectively identifying such cells. We assessed this possibility in non-diabetic recipients with the HLA-DRB1^*^04:01 (DR0401) haplotype as others have shown that autoreactive T cells specific to the islet beta cell antigens are readily detected in such subjects (15). Here, we confirmed the efficacy of our approach by isolating a CD4 T cell that can cross-recognize an epitope from influenza A matrix protein (MP) and an immunodominant epitope within the autoantigen Glutamic Acid Decarboxylase 65 (GAD65) protein in PBMC of a DR0401 healthy subject.

## Materials and methods

### Human subjects

A total of 4 healthy subjects with regular annual seasonal influenza vaccination in the past 3 years but without any known recent influenza infection history within the past year were recruited for the study. Each subject carried at least one copy of the DR0401 allele. All subjects received a single dose of the 2014-2015 Quadrivalent influenza vaccine by intramuscular injection (Fluzone, Sanofi Pasteur, Swiftwater, PA). The influenza A strains in the 2014–2015 vaccine included A/California/7/2009 (H1N1) and A/Texas/50/2012 (H3N2). The A/California/7/2009 (H1N1) strain was also a seasonal influenza vaccine strain in the past 3 years. The study was approved by the BRI internal review board (IRB) and written consent was obtained from all participants.

### Islet beta cell antigenic peptides and influenza antigenic peptides

All self-antigen peptides within GAD65 (glutamic acid decarboxylase 65), IGRP (Islet-Specific Glucose-6-Phosphatase Catalytic Subunit-Related Protein), Zn T8 (Zinc Transporter 8), and PPI (preproinsulin) used in this study had been previously identified as DR0401 restricted T cell epitopes (15–17). Sequences of these peptides are listed in Supplementary Table I. For simplicity of reading in the text and figures, the names of these peptides were defined by abbreviation of a protein name followed by peptide number, e.g., GADp1 is GAD65 protein peptide 1.

All influenza peptides selected were identified as DR0401 restricted epitopes by TGEM (18). H1HA is hemagglutinin from the H1N1 strain A/California/2009, H3HA is hemagglutinin from the H3N2 strain A/Texas/50/2012 and MP is a matrix protein from A/California/2009. All these influenza related peptides are listed in Supplementary Table II. All the islet antigen peptides and influenza A peptides were purchased from Mimotopes (Clayton, Australia).

### *Ex vivo* T cell activation, CD154 enrichment, and T cell sorting

A modified CD154 up-regulation assay (8–11) was used to identify islet beta cell antigen or influenza antigen specific CD4+ T cells e*x vivo*. Briefly, 20 million freshly isolated human PBMC in 4 ml of T cell culture medium (RPMI 1640 containing 10% (v/v) of pooled human serum, 1% (v/v) of L-Glutamine, sodium pyruvate, and Penicillin/Streptomycin) were stimulated *in vitro* for 3 h with peptides (2 μg/ml each) in the presence of anti-CD40 (1 μg/mL; clone HB-14, Miltenyi Biotec, San Diego, CA). PBMC were then stained with anti- CD154-PE antibody (clone 5C8, Miltenyi Biotec, San Diego, CA) and enriched using anti-PE microbeads (clone PE4-14D10, Mitenyi Biotec, San Diego, CA) per manufacturer's instructions. Enriched cells were then antibody labeled with: (1) anti-CD3-V500 (clone SP34-2), anti-CD4-APC-H7 (clone RPA-T4) to define CD4+ T cells, (2) anti-CD45RO-FITC (clone UCHL1) to define memory T cells, (3) anti-CD38-V450 (clone HB7) to define *in vivo* activated memory T cells, (4) anti-CD69-APC (clone L78) to define recently activated cell, and (5) anti-CD14-PerCP (clone MΦ9)/anti-CD19-PerCP (clone Leu-12)/via-Probe for an exclusion or dump gating. All antibodies were purchased from BD Biosciences (San Diego, CA). Islet beta cell antigen responsive CD4+ T cells within the cultured/expanded influenza responsive T cells were identified by up-regulation of CD154 and CD69 on CD4+CD3+ T cells. The *in vitro* activated islet beta cell antigen specific T cells were identified as CD154+CD69+CD45RO+CD38+T cells. In post-influenza vaccinated subjects who presented significant numbers of CD154+CD69+CD45RO+CD38+ T cells, subjects were recalled the next day for additional blood withdraws, and 100 million cells were processed as above and CD154+CD69+CD45RO+CD38+ T cells were sorted by using a BD FACS Aria and expanded as oligo-clones.

### Expansion of antigen specific activated T cells

Sorted antigen specific T cells (identified based on surface expression of CD154+CD69+CD38+) were seeded into round bottom 96-well plate at ~6 cells/well, including 1.5 × 10^5^ irradiated allogenic PBMC as feeder cells in 200 μL of T cell culture medium and 1 μg/ml of PHA (Fisher Scientific, Waltham, MA). Next day, each well was supplemented with 40 IU (in 10 μL of TCM) of recombinant human IL-2 (Sigma-Aldrich, St. Louis, MO). After 7–10 days culture at 37^0^C, 5% CO_2_, expanded T cells became visible colonies in the 96-well plate. These T cell colonies were then transferred to the flat-bottom 96-well plate and fed with 100 μL of fresh TCM supplemented with 200 IU/mL of IL-2. When the T cells become confluent in the plate, the cells were split and fed with fresh TCM and IL-2, and eventually transferred to 48-well plate. Approximately 5–10 × 10^6^ T expanded cells were obtained for CD154 epitope mapping assays.

### Epitope mapping with CD154 upregulation assay

Once the T cells were successfully expanded they were rested for at least 3 days in T cell media (TCM) in the absence of IL-2 prior to antigen stimulation. T cells from each oligoclonal lines were washed and suspended at 0.5 × 10^6^/mL in TCM containing 1 μg/mL of CD40 blocking Ab. 10^5^ T cells in 200 μL from each line were stimulated with 3 different pools of Influenza peptides (H1HA peptide pool, H3HA peptide pool or MP peptide pool) or without peptide as negative control. Cells were stimulated for 3 h, and then stained with Abs against CD3-FITC, CD4-PerCP, CD69-APC, and CD154-PE for 10 min. After washing off the excessive Abs, the up-regulation of CD154 upon antigen stimulation was analyzed by flow cytometry. If an oligoclonal T cell line responded to the pooled Influenza peptide stimulation, a second round of CD154 based epitope mapping was performed with individual peptides contained in that influenza peptide pool. An identical approach was used to map islet antigen epitopes.

### V beta flow cytometry typing and V alpha and V beta sequencing

Putative cross reactive T cells being identified were further sorted as single cells and expanded for TcR analysis. To assess the clonality of the cross reactive T cells, T cell clones were stained with V beta specific Abs (TcR Vβ Repertoire Kit, Beckman Coulter, Inc.,) per manufacturer instructions. The V beta type was determined according to the reference staining pattern from manufacturer. The TcR V alpha and V beta sequences of T cell clones of interest were also determined by 5′-RACE based cloning strategy according to manufacturer description (Takara Bio USA). Briefly, RNA was extracted from T cells, first strand cDNA was synthesized using oligo dT and switching adaptor. Then customized oligos in both V beta and V alpha constant region were used to PCR the V beta chain and V alpha chain pairing with the universal primer for the switching adaptor. hTRAC primer: GATTACGCCAAGCTTgttgctccaggccacagcactgttgctc, hTRBC primer: GATTACGCCAAGCTTcccattcacccaccagctcagctccacg. The products were cloned into sequencing vector and 12 colonies from each chain were sequenced. The resulting sequencing data were analyzed for V beta and V alpha usage and CDR3 sequences with IMGT/V-QUEST (http://www.imgt.org/IMGT_vquest/share/textes/imgtvquest.html) (19–21).

### T cell clone proliferation

Standard ^3^H-thymidine incorporation assays were performed to evaluate stimulatory activity of each peptide as described before (15). Briefly, clones were co-cultured with DR0401+ irradiated feeder cells and stimulated with various concentrations of influenza peptide, islet antigen peptide, or no peptide (as a negative control) for 48 h and then pulsed with 1 μCi ^3^H-thymidine (Amersham Biosciences, Piscataway, NJ) for an additional 16 h. Uptake of ^3^H-thymidine was measured with a scintillation counter to assess proliferation.

## Results

### CD38 is upregulated on recently activated antigen-specific (CD154+/CD69+) T cells

**Figure 1:**
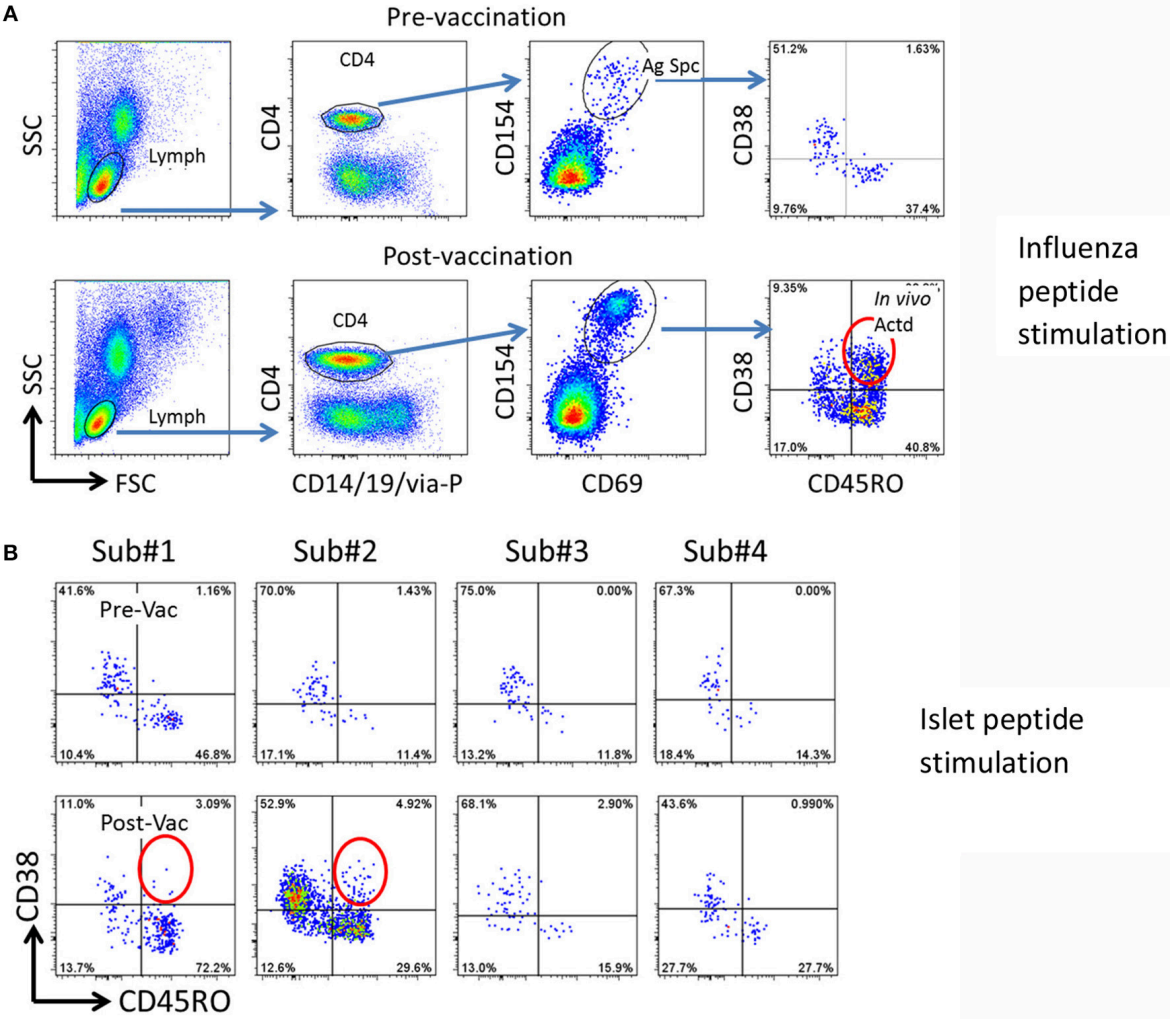
Strategy for detection of potential cross reactive T cells. **(A)** Gating procedure to define total and *in vivo* activated antigen specific T cells. Top panels, pre-vaccination, bottom panels, 14 days post-vaccination. PBMCs (from subject #2) were stimulated with 27 Influenza A peptides for 3 h. The total and *in vivo* activated Influenza A specific T cells were determined by the following gating strategy. (1) Lymphocytes (Lymph) were gated based on forward and side scatters (FSC and SSC). (2) CD4 T cells were gated by expression of CD14/CD19/via-Probe markers as dump gate. (3) Antigen specific (Ag Spc) T cells were determined by CD154 and CD69 up-regulation. (4.) *In vivo* activated (*In vivo* Actd) influenza A reactive T cells were defined by expression of CD38+ in the CD45RO+CD154+CD69+ subset. **(B)** Detection of islet antigen cross reactive T cells. Top panels, pre-vaccination, bottom panels, 14 days post-vaccination. PBMCs from four subjects were stimulated with DR0401 restricted islet antigen specific peptides. CD154 positive T cells were gated and analyzed as described in **(A)**. Subject #1 and #2 were identified as candidates to isolate cross-reactive T cells because of the emerging recently activated islet antigen specific CD45RO+CD38++ T cells (circled).

CD154 upregulation on CD4+ T cells after a 3 h influenza peptide stimulation of otherwise un-manipulated PBMC was used to identify influenza antigen specific CD4+ T cells. As previously reported (11), we first confirmed that CD38 was not upregulated in memory (CD45RO+) influenza specific T cells in PBMC during the 3 h *in vitro* stimulation in the CD154 upregulation assay (Figure 1A upper panel). Comparing CD38 expression in influenza specific CD4+/CD45RO+ memory T cells in pre and 14 days post influenza vaccination samples, we observed that CD38 expression was only present in the post vaccination samples (Figure 1A and Figure S1). This data confirmed that CD38+ can be used as a marker to identity antigen specific T cells (marked by CD154+CD69+) that have been recently activated *in vivo*.

### CD38 is upregulated on self-antigen reactive T cells following influenza vaccination

Using this approach with samples from 4 DR0401 healthy subjects, we then evaluated whether beta cell antigen specific CD38+ CD45RO+ T cells increased between the pre-vaccinated and 14 day post-influenza vaccinated samples. To address this question, PBMC from pre and post vaccinated individuals were stimulated with a peptide pool comprised of 24 DR0401 restricted antigenic peptides derived from islet beta cell antigens, GAD65, IGRP, ZnT8, and a modified insulin peptide (Supplementary Table I) (15–17). All these selected beta cell peptides were established DR0401 restricted T cell epitopes. An increase in frequency of CD154+CD69+CD45RO+CD38+ islet beta cell antigen specific CD4+ T cells was observed in subjects #1 and #2 14 days after influenza vaccination (Figure 1B). The frequency of CD38+CD45RO+ islet antigen specific cells were estimated to be 2.6/10^6^ and 3.5/10^6^ CD4+ T cells in subjects #1 and #2, respectively.

### Identification and epitope specificity of self- and viral-antigen cross reactive T cells

Subjects (#1 and #2) having shown an increase in CD45RO+CD38+ islet beta cell antigen specific T cells at 14-day post vaccination were recalled the next day after initial screening. A hundred million PBMC (20 × 10^6^ per well for 5 wells in a 6-well plate) were stimulated with 24 peptides derived from islet beta cell antigens. Activated islet beta cell antigen (CD154+CD69+CD45RO+CD38+) T cells were sorted for the generation of oligoclonal lines. For subject 1, 5 cells were cloned and expanded, but we failed to identify a cross-reactive clone with the procedure described below.

From subject #2, 109 *in vivo* activated (following vaccination) beta cell antigen specific T cells were sorted. These T cells were seeded in a round bottom 96-well plate at ~6 cells/well for a total of 18 wells in the presence of irradiated feeder cells. The T cells were stimulated with PHA, and expanded, yielding 18 oligoclonal lines with 5 to 10 million total cells each. As these cells were isolated after islet beta cell antigen stimulation, they should be highly enriched for beta cell antigen specific T cells.

**Figure 2:**
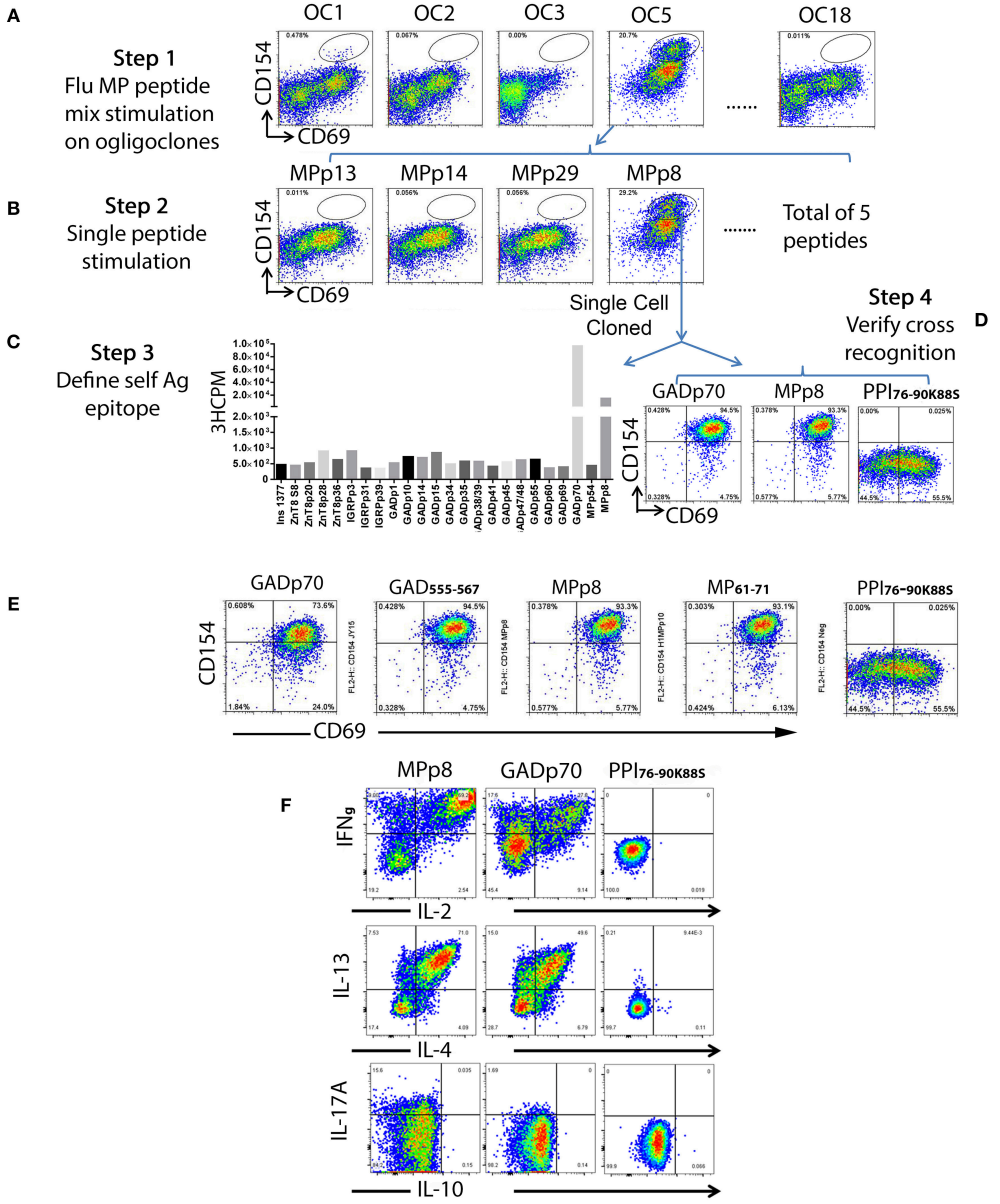
Detection of islet beta cell and Influenza A antigen cross reactive T cells and identification of epitope specificity. A total of one hundred and nine of beta cell antigen specific CD45RO+CD38++ T cells from subject #2 were sorted at 6 cells/well in 96-well plate and stimulated with PHA for expansion. All oligoclones (total of 18) were grown successfully. After expansion, each oligoclone was stimulated with 3 sets of Influenza antigenic peptide mixtures (i.e., H1HA, H3HA, and MP). **(A)** Oligoclone #1 and #5 contained a sub-population of T cells that response to the Influenza A MP peptide set stimulation. **(B)** Oligoclone #5 was stimulated with individual peptide from the MP peptide set. Oligoclone #5 contained a sub-population of T cells that response to the stimulation of Influenza MPp8 peptide. **(C)** Determining the cross-recognized peptide. The Influenza MPp8 peptide responding T cells were sorted out and expanded again. The T cell cross-recognition to islet beta cell antigen was defined by stimulation with individual islet beta cell antigenic peptides by ^3^H-thymidine incorporation assay. These purified T cells proliferated vigorously in respond to the GADp70 and MPp8 peptide stimulation. **(D)** Confirming the cross-recognition. Expanded purified T cells were stimulated with GADp70, MPp8 and a modified insulin peptide *in vitro* for 3 h, the up-regulation of CD154 and CD69 were compared. **(E)** Identifying the minimum peptide sequences that the cross reactive T cell clone recognizes. Cross reactive T cell clone was stimulated with either 20 aa of GADp70 and its corresponding 13 aa peptide GAD_555−567_ or MPp8 peptides and its corresponding 11 aa peptide MP51-61. An irrelevant modified insulin peptide was used as negative control. **(F)** Cytokine secretion profiles of cross reactive T cells in respond to the peptide stimulation. T cells were stimulated with either MPp8, GADp70 or irrelevant modified insulin peptides in the presence of Brefidin A. After stimulation, the cells were fixed, permeablized and stained with antibodies specific to different cytokines.

To identify T cells that are cross-reactive to Influenza antigen, each oligoclonal line underwent a 3 h stimulation CD154 up-regulation assay with influenza H1HA, H3HA, or MP pooled peptides mixtures (Supplementary Table II). Though responses of the oligoclonal lines to H1HA and H3HA were negative, both oligoclones #1 and #5 contained a subpopulation of MP antigen responsive T cells (Figure 2A, step 1). T cells from oligoclone #5 were stimulated with individual MP antigenic peptides and MPp8 peptide was identified as the antigenic peptide (Figure 2B, step 2). Similarly, a tiny fraction of T cells in oligoclone #1 also show cross reactivity to MPp8 (data not shown). Influenza MPp8 specific T cells from oligoclone #5 were then single cell FACS sorted and expanded with PHA again. A total of 6 T cell clones were isolated. All these clones expressed TRB5-1 (see below). The islet antigen specificity of one of these MPp8 reactive T cell clones was determined by T cell proliferation assays with the panel of 24 selected islet peptides. Proliferation assays revealed that the clone recognized both GADp70 and MPp8 peptides (Figure 2C). To confirm the results of the proliferation assay, the T cell clone was stimulated with GADp70, MPp8, and an Insulin peptide, respectively. As shown in Figure 2D, the T cell clone responded in a CD154 upregulation assay to both GADp70 and MPp8 stimulation but not to a control Insulin peptide. We further narrowed down the cross-reactive epitopes using two short peptides, a 13 aa GAD_555−567_ (NF**F**RM**V**I**S**NP**A**AT) and an 11 aa MP_61−71_ (G**F**VF**T**L**T**VP**S**E) to stimulate the T cell clone (the bolded letters denote the predicted HLA-DR0401 anchoring residues). As shown in Figure 2E, both short peptides were effective in activating the T cell clone in the CD154 assay. Of note, both peptides contain typical DR0401 binding motifs. However, both DR0401/GAD_555−567_ and DR0401/MP_61−71_ tetramers were incapable of staining this T cell clone, suggesting that the TCR had low avidity for both the DR0401/GAD_555−567_ and DR0401/MP_61−71_ complexes (data not shown). To evaluate whether the two peptides will stimulate the T cell clone to secrete a different set of cytokines, we stimulated the clone with MPp8 and GADp70 peptides. Both peptides elicited similar cytokine secretion patterns, i.e., secretion of IFNγ, IL-2, IL-13, and IL-4, but not IL10- and IL-17A, a typical Th0 cytokine secretion profile (Figure 2F).

### TcR usage, HLA class II restriction, and cytokine profiles of cross reactive T cells

**Figure 3:**
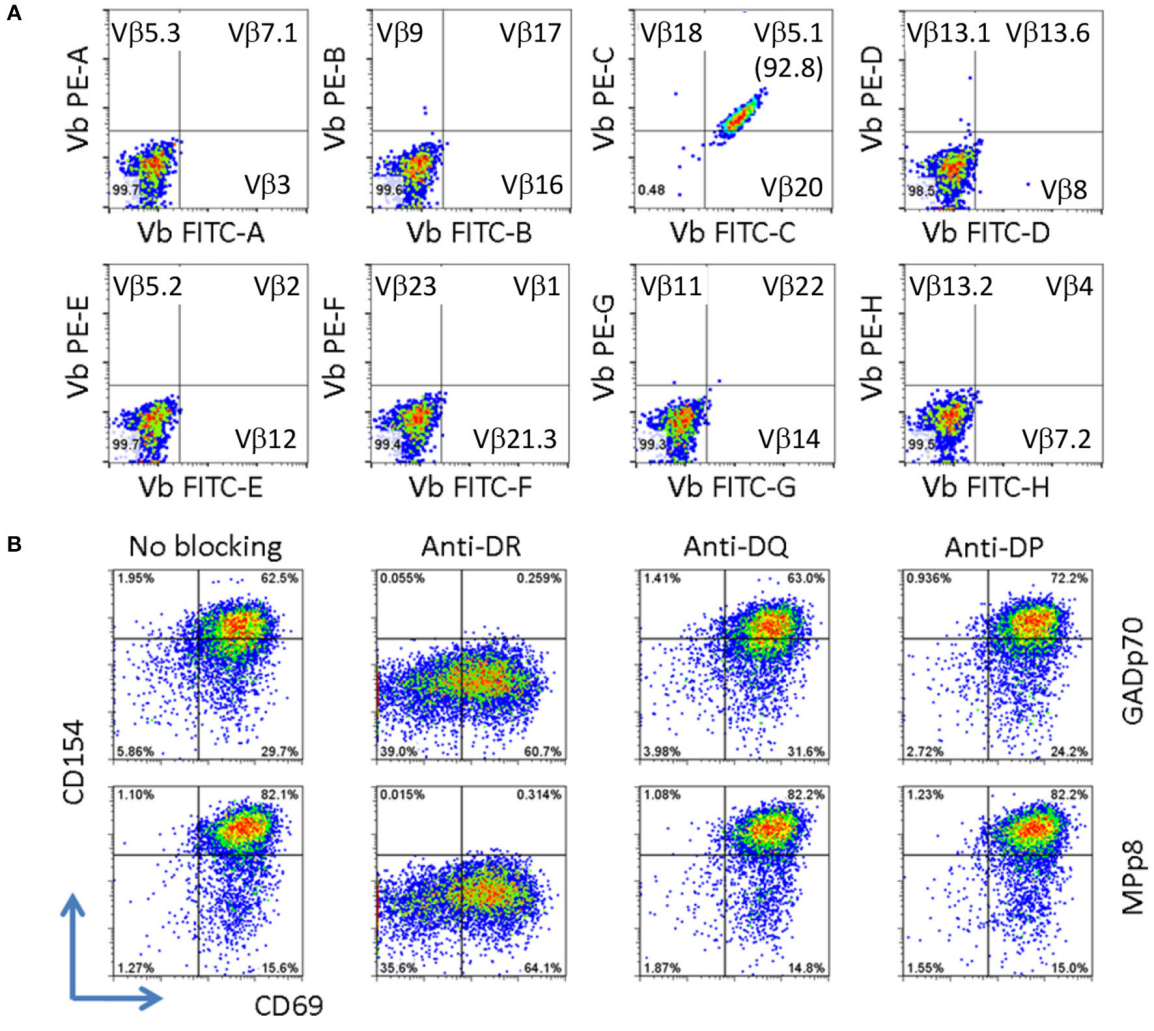
Clonality and HLA restriction of cross reactive T cell clone. **(A)** V beta typing of cross reactive T cell clone. The V beta typing of TcR was interpreted based on the antibody staining pattern provided by the TcR Vβ Repertoire Kit. **(B)** Determining HLA restriction of T cell clone. The T cells were stimulated with either GADp70 or MPp8 peptide in the absence or presence of different HLA class II blocking Abs. The restriction HLA for the peptide was identified by the lack of response when the corresponding HLA blocking antibody was present in the stimulation.

The clonality of the GAD65p70/Influenza MPp8 T cell clone was confirmed by both TcR V beta antibody typing and TcR sequencing. Using a panel of TcR V beta typing antibodies we found the clone only expressed TRB5-1 (Figure 3A). The TcR sequencings of V alpha and V beta obtained by 5′-RACE, also demonstrated that the cross reactive clone has only one TcR alpha chain (Homsap TRAV9-2^*^01, TRAJ53^*^01, Junction AA: CALSDPNGGSNYKLTF) and one TcR beta chain (Homsap TRBV5-1^*^01, TRBD2^*^01, TRBJ2-3^*^01, Junction AA: CASSSFPLAGGPTDTQYF), determined by IMGT/V-QUEST analysis (20, 21) (Table 1).

**Table 1:** TcR CDR3 Region sequence.

**TcR alpha chain**	— AV9-2^*^01—|							| — — — — — - J53^*^01 — — — — — — -													
AV9-2^*^01/J53^*^01	C	A	L	S	D	P	N	–	–	G	G	S	N	Y	K	L	T	F			
								*N*	*S*
**TcR beta chain**	— BV5-1^*^01 — -|								| — – D2^*^01 — –|						| — – J2-2^*^01 — — — –						
BV5-1^*^01/D2^*^01/J2-3^*^01	A	S	S	–	S	S	F	P	–	L	A	G	G	P	–	T	D	T	Q	Y	F
				*L*					*G*						*S*

*Amino acid residues that are germline coded, but removed upon recombination are shown italicized*.

Besides the mechanism of sequence dependent molecular mimicry, cross-activation can also be induced by structural similarity in the context of peptide/MHC complex (22, 23). This scenario can occur for two sequence unrelated peptides presented on two different HLA molecules. As such we evaluated whether the T cell clone recognizes GADp70 and MPp8 peptides in the context of two different HLA molecules. To test this possibility, the T cell clone is stimulated with GADp70 and MPp8 peptides in the presence of HLA-DR (L243), DQ (SPVL3) and DP (B7/21) blocking antibodies (24–26). As show in Figure 3B, blocking DR abolished peptide stimulation activities, while no effect when blocking DQ and DP molecules. Therefore, HLA-DR is the restriction element for presenting both peptides (Figure 3B).

## Discussion

Many hypotheses have been proposed for the activation of autoreactive T cells by infectious agents (27, 28) and include: molecular mimicry (29), bystander activation (30, 31), entry of T cells to immune privilege sites that lead to the release of sequestered autoantigens (32) and dual TcR on T cells (33). In molecular mimicry microbial antigens share sequential or structural homology with an autoantigen. T cells activated by epitopes derived from bacterial or viral antigen fragments during infection or vaccination can subsequently attack target tissue that expresses a homologous protein sequence. In studying molecular mimicry in T1D, extensive amino acid sequence homology between Coxsackie virus protein P2C 30-50 and GAD65 247-280 has been reported as a possible source of mimicry (34). Similarly, T cell antigen molecular mimicry has also been found in human IA-2 805-817 and rotavirus VP 40-52 epitopes where five out of the 9 aa in the core T cell epitopes are identical (35). Subsequently, it was demonstrated that inoculation of rotavirus into weaning mice by oral gavage led to transient hyperglycemia (36). Despite these observations, extensive sequence homology between viral antigens and islet antigens is relatively uncommon. In the current study, we developed a strategy to detect *in vivo* activated autoreactive T cells that are potentially activated during natural vaccination. First, we exploited the observation that memory CD4+ expresses CD38 upon reactivation for 4 to 8 weeks. These allowed us to detect islet antigen specific T cells that are potentially cross-activated by influenza antigens following vaccination. Second, we selected previously identified immunodominant DR0401 restricted epitopes for islet antigens (24 instead of the 172 peptides that would have required to cover all GAD65, ZnT8, IGRP, and Preproinsulin peptides if a complete overlapping peptide strategy had been used) (15–17) and HA and MP influenza antigens (27 peptides instead of at least two thousand of peptides needed to cover all the viral proteins for four influenza strains contained in Fluzone quadrivalent vaccine) in the study. This strategy significantly reduced down-stream work-load in antigen specific T cell screening and their corresponding epitope identification. Third, our newly developed modified CD154-epitope mapping assay (11) provided the technical possibility to accomplish epitope identification.

Using this technique we observed that influenza vaccination can increase the detection of recently activated islet antigen specific GAD65 T cells and that some of this increase is due to T cell cross reactivity to an Influenza MP Protein. Fine epitope mapping analysis on a cross-reactive clone revealed that the T cell recognizes both influenza A MP_61−71_ and GAD65_555−567_ peptides in a MHC DR restricted manner. GAD65_555−567_ (NF***F***RMVISNP***A***AT, bold residues defined as P1 and P9 anchor residues) peptide has been identified as a DR0401 restricted T cell epitope within a naturally processed GAD65_552−572_ epitope (37), while MP_61−71_ (G***F***VFTLTVP***S***E) is an immunodominant DR0401 restricted T cell epitope (38). However, since the donor is DR0401 and DRB1^*^13:01 (DR1301), we cannot rule out the possibility that DR1301 or DRB4 may also be presenting these epitopes. Under the scenario of DR0401 presentation and predicted MHC-binding pockets, only 2 out of 9 residues of the core sequence of these 2 peptides are identical. Such an observation infers that cross recognition can occur with minimal amino acid sequence homology. In fact, this type of cross-recognition has been observed elsewhere. For example: (1) CD4 T cells recognize acetylated N-terminal nonapeptide of mouse MBP (Ac1-11) autoantigen only require two amino acids for activation (39); (2) a CD4 T cell clone induces B cell lymphoma apoptosis in a dose-dependent and peptide-specific manner for two distinct endogenous peptides, Hb (64–76) and moth cytochrome c (93–103) (40), (3) T cell hybridomas recognize allelic forms of human alpha 1-antitrypsin (hAAT) of PiM 205–220 and PiM 335–350 peptides with only two out of 16 amino acid identity (41), and (4) a human CD4 T cell clone isolated from a Graves disease thyroid infiltrate can recognize two minimal peptides of peptide thyroid peroxidase (TPO_536−547_ and TPO_539−550_) which bind to DQ6 with distinct binding registers (22).

**Figure 4:**
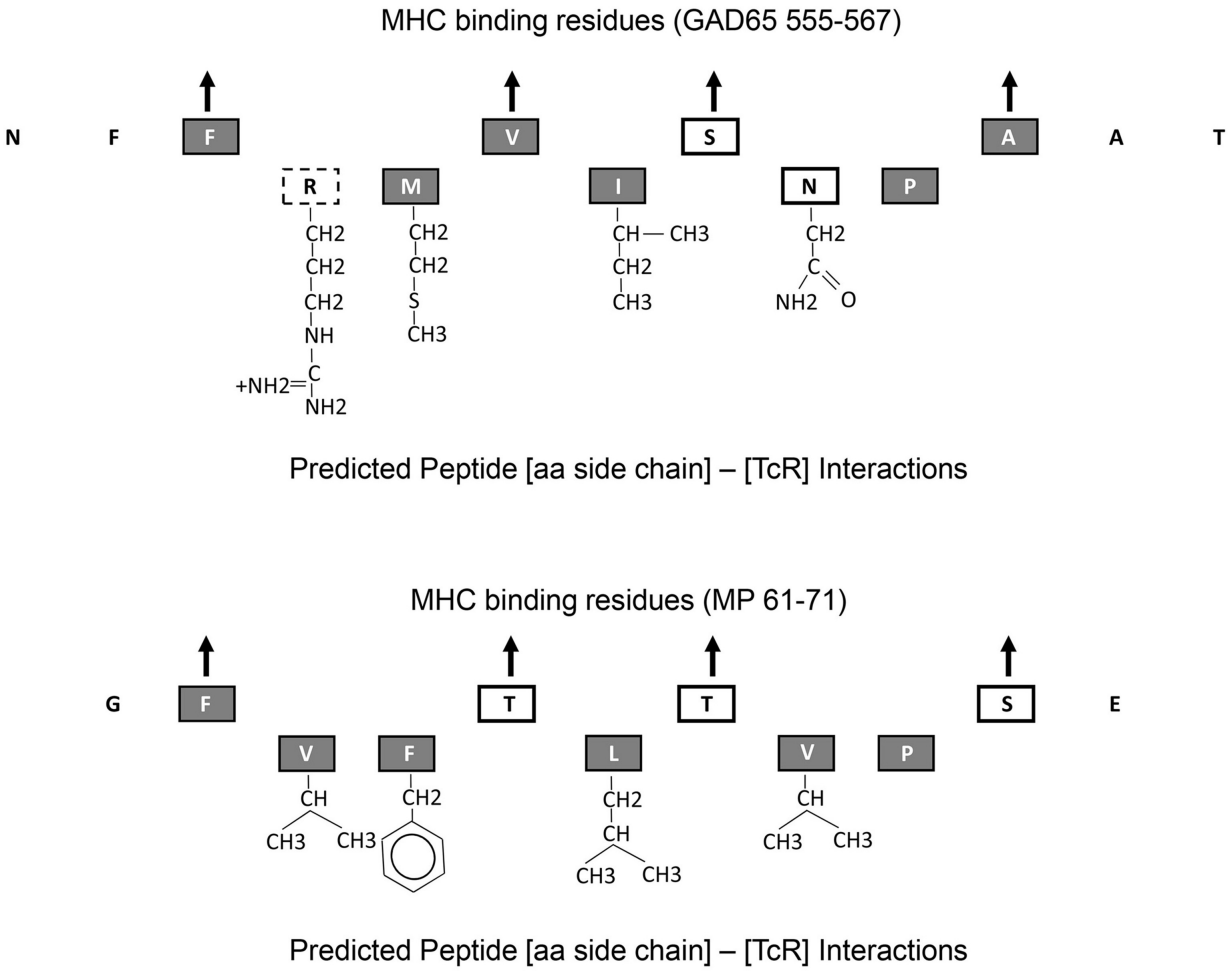
Predicted MHC-binding and TcR interacting amino acid residues within GAD65_555−567_ and MP_61−71_. Up point arrows denote predicted amino acid residues within the peptide that bind to the MHC. Chemical structure of those amino acid side chains that interact with the TcR are shown. Amino acid letters are coded as follows: gray-filled boxes—non-polar side chains, black line boxes—polar side chains, and dotted line box—charged polar chain.

All these observations suggest that MHC-binding peptides that cross activate autoreactive T cells with a single TcR alpha and a single TcR beta chain can have minimal aa overlap. GAD65_555−567_ and MP_61−71_ epitopes have minimum sequence homology, yet both bind the DR0401 MHC by utilizing relative well conserved residues at the four MHC pocket binding sites, P1, P4, P6, and P9. However, the aa side chains that interact with the TcR exhibit fairly large structural differences (Figure 4). Only one of the 5 aa side chains that are predicted to interact with the TcR is identical (P8). The P5 residues for both peptides, I and L are well conserved. However, aa side chain differences at P2 involve a small non-polar (Valine) to a large charged polar group (Arginine) and at P7 a change from a non-polar (Valine) to a polar group (Asparagine). One way to accommodate such large structural changes and still be able to interact and activate the same TcR would be to alter the [MHC]-[peptide]-[TcR] axial orientation. The natural [MHC]-[peptide]-[TcR] axial orientation (the docking angle between the MHC-bound peptide axis and the TcR) has been shown by crystallography studies to vary by over 60 degrees (42). Such axial changes have been observed by crystallography of a single TcR in the context of two different MHC molecules where one binds a self-antigen and the other a foreign antigen (43). Crystallography studies will be required to confirm whether such changing in docking orientation occurs in the TCR/pMHCs under the current study.

The absence of significant aa sequence homology between the two peptides identified here could be taken to suggest that cross activation of autoreactive T cells by a foreign antigen derived peptides may be a more common phenomenon than has been previously perceived. Previous studies in autoimmune diabetes have been frequently focused on molecular mimicry by highly homologous peptides derived from pathogens such as in rotaviruses and coxsackie viruses (34, 35). Our current study suggests that many viruses and bacteria can potentially activate autoreactive T cells through molecular mimicry in the absence of obvious amino acid sequence homology. If such degenerate TcR recognition is common, peptides derive from viruses and gut flora could activate autoreactive T cells in the islet milieu and result in pathogenic autoimmunity to beta cells.

The current study focuses on influenza vaccination and islet reactive T cells. Through our new methodology, we have clearly documented the presence of cross-reactive memory T cells in a limited number of subjects. Although the size of the cohort in the current study was insufficient to draw generalized conclusions about the role that such cells might play in autoimmunity, the work provided a rationale for additional studies designed to examine whether vaccination against influenza or other infectious pathogens can activate cross-reactive T cells that are relevant in TID (or in other autoimmune diseases). Based on our findings, assessing the biological significance of this type of cross activation in the initiation, development and prevention of type 1 diabetes and other autoimmune diseases is an appropriate question, which will require a carefully designed sample set to address.

## Ethics statement

In compliance with the human subject guidelines set by the Declaration of Helsinki, this study was approved by the Benaroya Research Institute Institutional Review Board Human Subjects Review Committee (IRB). All subjects gave written informed consent in accordance with the Declaration of Helsinki. Vulnerable populations, for example minors, persons with disabilities, were excluded for the study.

## Author contributions

JY and WK designed the experiments. JY and LJ performed the experiments. EJ provided the samples. JY, JG, DK, and WK analyzed the data. JY and WK wrote the manuscript. JY, LJ, EJ, JG, and DK have all read and revised/updated the manuscript as needed.

### Conflict of interest statement

The authors declare that the research was conducted in the absence of any commercial or financial relationships that could be construed as a potential conflict of interest.
