# Supplementary material for: A Novel Approach of Identifying Immunodominant Self and Viral Antigen Cross-Reactive T Cells and Defining the Epitopes They Recognize
Source: Front Immunol. 2018 Dec 3;9:2811. doi: 10.3389/fimmu.2018.02811 (PMC6298415; doi:10.3389/fimmu.2018.02811)
Supplement: Supplementary file 1 [file Table_1.DOCX]

| Peptide | Sequence | Position |
| --- | --- | --- |
| GAD65 |  |  |
| GADp1 | MASPGSGFWSFGSEDGSGDS | 1 - 20 |
| GADp10 | CACDQKPCSCSKVDVNYAFL | 73 - 92 |
| GADp14 | RPTLAFLQDVMNILLQYVVK | 105 - 124 |
| GADp15 | DVMNILLQYVVKSFDRSTKV | 113 - 132 |
| GADp34 | KGMAALPRLIAFTSEHSHFS | 265 - 284 |
| GADp35 | LIAFTSEHSHFSLKKGAAAL | 273 - 292 |
| GADp38 | DSVILIKCDERGKMIPSDLE | 297 - 316 |
| GADp39 | DERGKMIPSDLERRILEAKQ | 305 - 324 |
| GADp41 | EAKQKGFVPFLVSATAGTTV | 321 - 340 |
| GADp45 | ICKKYKIWMHVDAAWGGGLL | 353 - 372 |
| GADp47 | GGLLMSRKHKWKLSGVERAN | 369 - 388 |
| GADp48 | HKWKLSGVERANSVTWNPHK | 377 - 396 |
| GADp55 | YDLSYDTGDKALQCGRHVDV | 433 - 452 |
| GADp60 | KCLELAEYLYNIIKNREGYE | 473 - 492 |
| GADp69 | VSYQPLGDKVNFFRMVISNP | 545 - 564 |
| GADp70 | KVNFFRMVISNPAATHQDID | 553 - 572 |
| IGRP |  |  |
| IGRPp3 | KDYRAYYTFLNFMSNVGDPR | 17-36 |
| IGRPp31 | KWCANPDWIHIDTTPFAGLV | 241-260 |
| IGRPp39 | QLYHFLQIPTHEEHLFYVLS | 305-324 |
| ZnT8 |  |  |
| ZnT S8 | MEFLERTYLVNDKAAKMYAF | 1-20 |
| ZnTp20 | GHNHKEVQANASVRAAFVHA | 202-221 |
| ZnTp28 | ILKDFSILLMEGVPKSLNYS | 266-285 |
| ZnTp36 | VRREIAKALSKSFTMHSLTI | 330-319 |
| Preproinsulin |  |  |
| PPI 1377 | SLQPLALEGSLQKSG | PPI76-90^R88S^ |

**Supplementary Table I. DRB1*04:01 restricted islet antigen specific T cell epitopes.**
