# Supplementary material for: A Novel Approach of Identifying Immunodominant Self and Viral Antigen Cross-Reactive T Cells and Defining the Epitopes They Recognize
Source: Front Immunol. 2018 Dec 3;9:2811. doi: 10.3389/fimmu.2018.02811 (PMC6298415; doi:10.3389/fimmu.2018.02811)
Supplement: Supplementary file 2 [file Table_2.DOCX]

| **Peptide** | **Sequence** | **Position** |
| --- | --- | --- |
| Hemagglutinin A/California/2009 |  |  |
| H1HAp32 | TLVEPGDKITFEATGNLVVP | 249 - 268 |
| H1HAp34 | LVVPRYAFAMERNAGSGIII | 265 - 284 |
| H1HAp42 | LRLATGLRNIPSIQSRGLFG | 329 - 348 |
| H1HAp50 | TNKVNSVIEKMNTQFTAVGK | 393 - 412 |
| H1HAp51 | EKMNTQFTAVGKEFNHLEKR | 401 - 420 |
| H1HAp56 | ELLVLLENERTLDYHDSNVK | 441 - 460 |
| H1HAp66 | KLESTRIYQILAIYSTVASS | 521 - 540 |
| H1HAp16 | QLSSVSSFERFEIFPKTSSW | 121 - 140 |
| H1HAp17 | ERFEIFPKTSSWPNHDSNKG | 129 - 148 |
| H1HAp27 | YQNADTYVFVGSSRYSKKFK | 209 - 228 |
| H1HAp29 | KKFKPEIAIRPKVRDQEGRM | 225 - 244 |
| H1HAp33 | ITFEATGNLVVPRYAFAMER | 257 - 276 |
| H1HAp58 | SNVKNLYEKVRSQLKNNAKE | 457 - 476 |
| Hemagglutinin A/Texas/50/2012 |  |  |
| H3HAp3 | HHAVPNGTIVKTITNDRIEV | 17-36 |
| H3HAp5 | RIEVTNATELVQNSSIGEIC | 33-52 |
| H3HAp6 | ELVQNSSIGEICDSPHQILD | 41-60 |
| H3HAp7 | GEICDSPHQILDGENCTLID | 49-68 |
| H3HAp13 | CYPYDVPDYASLRSLVASSG | 97-116 |
| H3HAp34 | SSIMRSDAPIGKCNSECITP | 265-284 |
| H3HAp38 | VNRITYGACPRYVKQSTLKL | 297-316 |
| H3HAp39 | CPRYVKQSTLKLATGMRNVP | 305-324 |
| H3HAp42 | RGIFGAIAGFIENGWEGMVD | 329-348 |
| Matrix Protein A/California/2009 |  |  |
| MPp8 | KGILGFVFTLTVPSERGLQR | 57 - 76 |
| MPp13 | VKLYKKLKREITFHGAKEVS | 97 - 116 |
| MPp14 | REITFHGAKEVSLSYSTGAL | 105 - 124 |
| MPp15 | KEVSLSYSAGALASCMGLIY | 113-132 |
| MPp22 | TNPLIRHENRMVLASTTAKA | 169-188 |

**Supplementary Table II. DRB1*04:01 restricted influenza A antigen specific T cell epitopes.**
