# Supplementary figures and images for: A Novel Approach of Identifying Immunodominant Self and Viral Antigen Cross-Reactive T Cells and Defining the Epitopes They Recognize
Source: Front Immunol. 2018 Dec 3;9:2811. doi: 10.3389/fimmu.2018.02811 (PMC6298415; doi:10.3389/fimmu.2018.02811)

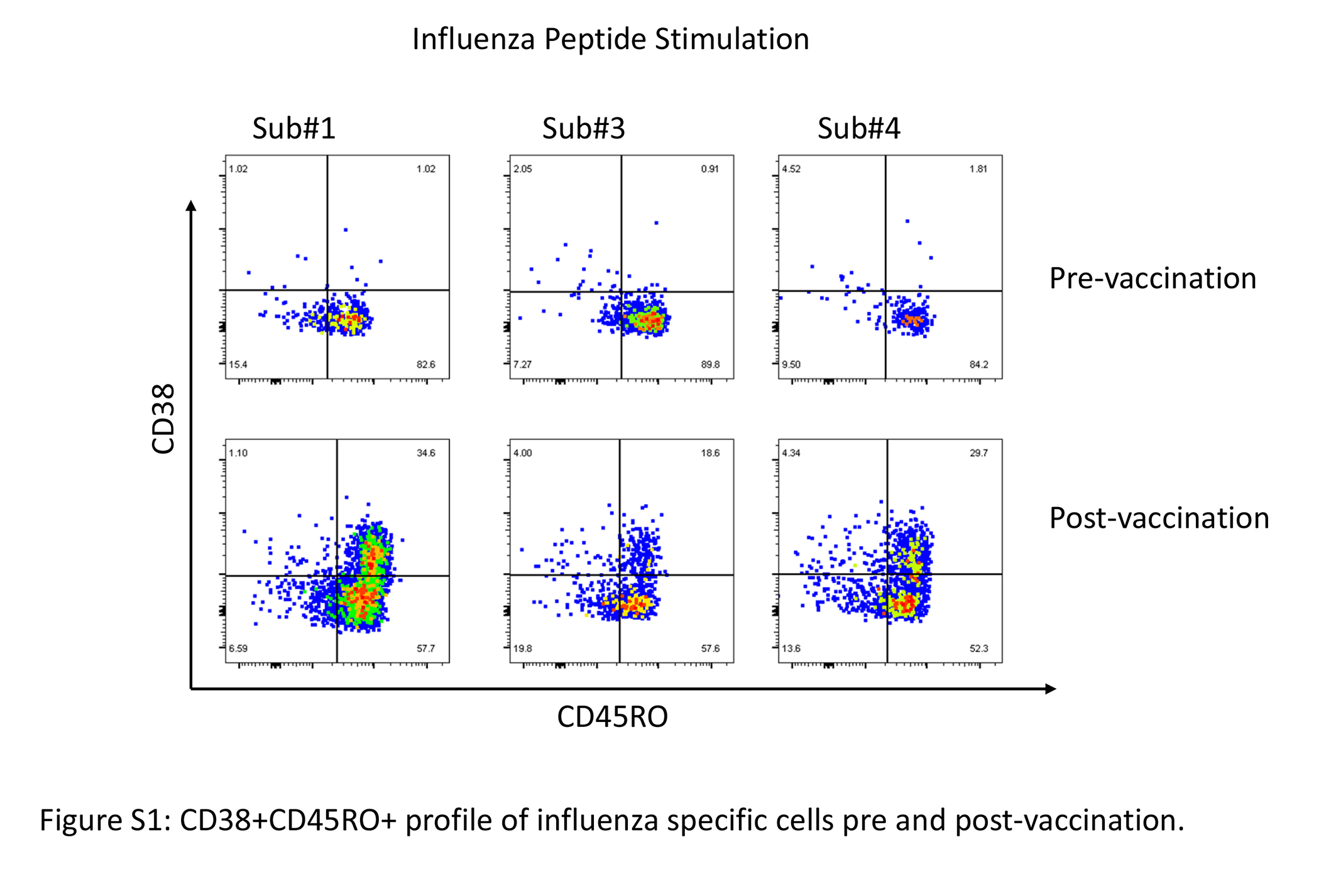

Supplement: Supplementary file 3 [file Image_1.JPEG]
